# Effect of Nitrogen Sources on Omega-3 Polyunsaturated Fatty Acid Biosynthesis and Gene Expression in *Thraustochytriidae* sp.

**DOI:** 10.3390/md18120612

**Published:** 2020-12-01

**Authors:** Siting Li, Zhangli Hu, Xuewei Yang, Yan Li

**Affiliations:** 1 Guangdong Technology Research Center for Marine Algal Bioengineering, Guangdong Key Laboratory of Plant Epigenetics, College of Life Sciences and Oceanography, Shenzhen University, Shenzhen 518060, China; siting.li@aut.ac.nz (S.L.); huzl@szu.edu.cn (Z.H.); 2 Faculty of Health and Environmental Sciences, School of Science, Auckland University of Technology, Auckland 1142, New Zealand; 3 Shenzhen Key Laboratory of Marine Biological Resources and Ecology Environment, Shenzhen Key Laboratory of Microbial Genetic Engineering, College of Life Sciences and Oceanography, Shenzhen University, Shenzhen 518055, China; 4 Longhua Innovation Institute for Biotechnology, Shenzhen University, Shenzhen 518060, China

**Keywords:** nitrogen source, *Thraustochytriidae* sp, n-3 polyunsaturated fatty acids, elongation and unsaturation, reference genes

## Abstract

The molecular mechanism that contributes to nitrogen source dependent omega-3 polyunsaturated fatty acid (n-3 PUFA) synthesis in marine oleaginous protists *Thraustochytriidae* sp., was explored in this study. The fatty acid (FA) synthesis was significantly influenced by the supplement of various levels of sodium nitrate (SN) (1–50 mM) or urea (1–50 mM). Compared with SN (50 mM) cultivation, cells from urea (50 mM) cultivation accumulated 1.16-fold more n-3 PUFAs (49.49% docosahexaenoic acid (DHA) (*w*/*w*, of total FAs) and 5.28% docosapentaenoic acid (DPA) (*w*/*w*, of total FAs)). Strikingly higher quantities of short chain FAs (<18 carbons) (52.22-fold of that in urea cultivation) were produced from SN cultivation. Ten candidate reference genes (RGs) were screened by using four statistical methods (geNorm, NormFinder, Bestkeeper and RefFinder). MFT (Mitochondrial folate transporter) and NUC (Nucleolin) were determined as the stable RGs to normalize the RT-qPCR (real-time quantitative polymerase chain reaction) data of essential genes related to n-3 PUFAs-synthesis. Our results elucidated that the gene transcripts of delta(3,5)-delta(2,4)-dienoyl-CoA isomerase, enoyl-CoA hydratase, fatty acid elongase 3, long-chain fatty acid acyl-CoA ligase, and acetyl-CoA carboxylase were up-regulated under urea cultivation, contributing to the extension and unsaturated bond formation. These findings indicated that regulation of the specific genes through nitrogen source could greatly stimulate n-3 PUFA production in *Thraustochytriidae* sp.

## 1. Introduction

Fatty acids (FAs) are essential compounds that play significant roles in cellular structure, energy storage, physiological metabolism as well as genetic regulation [1]. Omega-3 polyunsaturated fatty acids (n-3 PUFAs) [2] have various health benefits such as healthy brain and eye development [3,4], cardiovascular disease prevention [5,6], anti-inflammatory [7], and anti-cancer effects [8]. Currently, marine oily fish is the main source of n-3 PUFAs, which has shown obvious drawbacks, including accumulated contaminants and unstable wild fish stocks, hence alternative sources are required to meet the increasing global market demand [9,10]. *Thraustochytrids*, heterotrophic eukaryotic marine protists, have attracted increasing attention for their significant lipid production capacities. Being considered as a sustainable alternative source of n-3 PUFAs, *Thraustochytrids* have advantages of high lipids contents, fast growth rate and easily large-scale fermentation [11]. For instance, maximum lipid yield (% dry weight basis) achieved were 40.5% and 49.4%, respectively, in *Schizochytrium* sp. S31 (ATCC) and *Schizochytrium* sp. DT3 [12]. Different fermentation strategies have been employed to improve lipid yield and FA profile, such as optimization in salinity, pH, dissolved oxygen, temperature, carbon source and nitrogen source [13,14]. Several desaturases and elongases participated in FAS pathway and synthase involved in PKS pathway have been identified [15].

Nitrogen is an indispensable nutrient affecting microalgal growth and lipid production [16,17,18]. Previous research has shown that nitrogen starvation can effectively increase lipid content in *Thraustochytrids*. *Aurantiochytrium* sp. strain T66, which accumulated the highest lipid content of 63% (*w*/*w*) of dry cell weight under the condition of nitrogen limitation combined with O_2_ limitation [19]. Ren, Sun, Zhuang, Qu, Ji and Huang [14] reported that in the fermentation of *Schizochytrium* sp. ABC101, limited nitrogen feeding increased lipid yield by 1.5-fold. With sodium nitrate addition, *Aurantiochytrium* sp. KRS101 cultivated in orange peel extract medium achieved 50.23% total fatty acid (TFA, % DCW) and 28.51% docosahexaenoic acid (DHA) [20]. When using urea as a nitrogen source, *Aurantiochytrium* sp. SD116 accumulated 71.09% TFA and 34.79% DHA [21]. However, the molecular mechanisms underlying the effect of nitrogen source on lipids production in remains unclear.

The reverse transcription real-time quantitative polymerase chain reaction (RT-qPCR) is a widely used technique for the determination of gene [22]. Reference genes (RGs) are of great importance to normalize data and assure accurate results of RT-qPCR. For each experimental condition, it is essential to validate the expression stability of RGs to avoid false results and/or erroneous interpretations [23]. To date, research about RGs in *Thraustochytrids* is limited. 18S rRNA was used as an internal RG of *Aurantiochytrium* sp. under low temperature conditions [24]. Chen et al. [25] also used 18S rRNA as an internal standard in *Schizochytrium* sp. S056 when glycerol is used as a carbon source in fermentation. ACT (actin) was set as a RG to validate genes involved in biosynthetic pathways of docosahexaenoic acid (DHA) and the ketocarotenoid astaxanthin in *Aurantiochytrium* sp. SK4 [26]. However, previous publications have indicated that housekeeping genes are not universally suitable RGs for various microorganisms and experimental conditions [27,28,29]. Currently the suitable RGs in *Thraustochytriidae* sp. PKU#Mn16 under nitrogen stress is lacking.

In this study, we investigated the effects of different nitrogen sources (e.g., sodium nitrate or urea) on the lipid production in *Thraustochytriidae* sp. PKU#Mn16. To further explore the molecular mechanism that contributes to nitrogen source dependent n-3 PUFAs synthesis in *Thraustochytriidae* sp. PKU#Mn16, several crucial genes (delta(3,5)-delta(2,4)-dienoyl-CoA isomerase, enoyl-CoA hydratase, fatty acid elongase 3, long-chain fatty acid acyl-CoA ligase, acetyl-CoA carboxylase and nitrate reductase) identified in our previous transcriptome studies were quantitated by using RT-qPCR. Due to the lack of available RGs for abovementioned experimental conditions, novel candidate genes were selected and evaluated to obtain the most suitable RGs for gene expression in *Thraustochytrids*. This study provides a better understanding of underlying mechanisms of the biosynthesis of n-3 PUFAs in *Thraustochytrids* cultivated under different nitrogen sources.

## 2. Results

### 2.1. Effects of Sodium Nitrate and Urea on the Growth of Thraustochytriidae sp. PKU#Mn16

**Figure 1:**
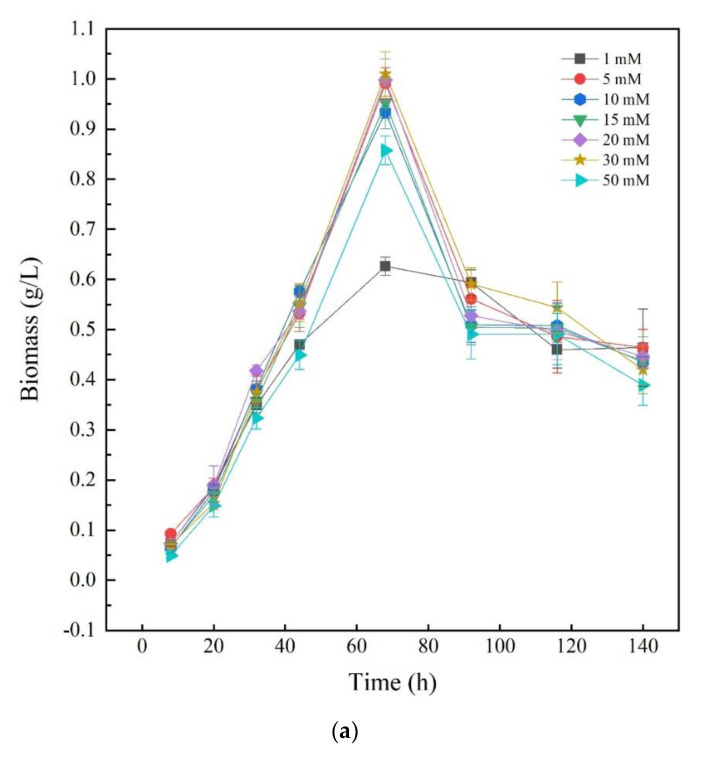

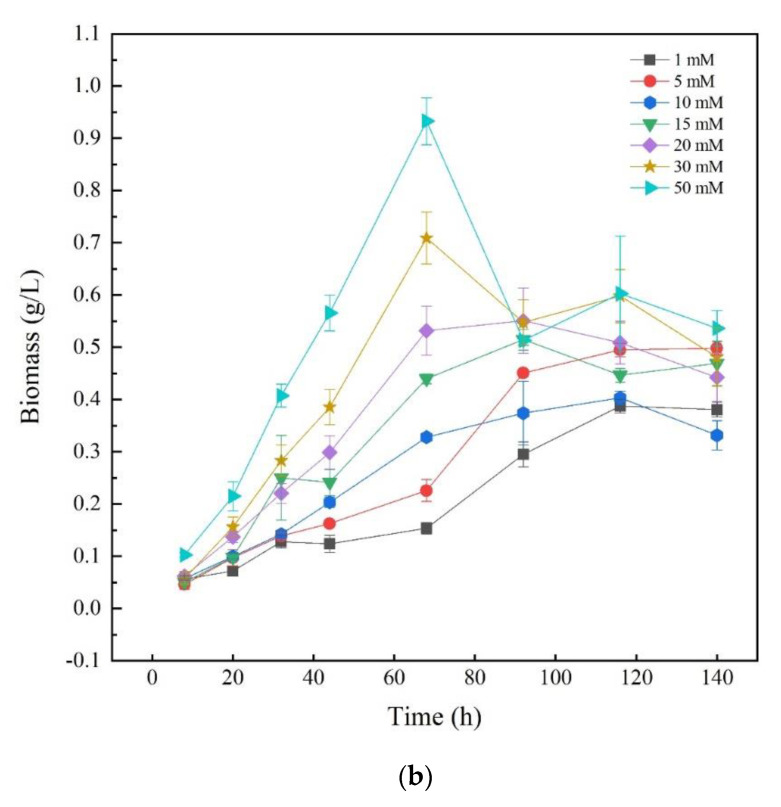
Growth of *Thraustochytriidae* sp. PKU#Mn16 cultivated with sodium nitrate (**a**) or urea (**b**) as nitrogen sources. All tests were performed in biological triplicate. Values were means ± standard deviation.

To investigate the effects of different nitrogen sources on the growth of *Thraustochytriidae* sp. PKU#Mn16, inorganic nitrogen source sodium nitrate (SN) and organic nitrogen source urea were chosen as the sole nitrogen source, respectively. Seven concentrations of N in the medium (1, 5, 10, 15, 20, 30, and 50 mM) were tested for *Thraustochytriidae* sp. PKU#Mn16 flask cultivations. As shown in Figure 1a, the growth trends of *Thraustochytriidae* sp. PKU#Mn16 were generally similar to each other for all the tested SN concentrations. Cell growth was relatively slow within the first 20 h, then entered the logarithmic phase with the time to peak biomass of 68 h, after which cell growth started to decline. At 68 h, the highest peak biomass (dry cell weight (DCW), 1.01 g/L) was observed in 30 mM SN cultivation, followed by 20 mM (1.00 g/L), 5 mM (0.99 g/L), 15 mM (0.95 g/L), 10 mM (0.93 g/L), 50 mM (0.86 g/L), and the lowest peak biomass occurred in 1 mM cultivation (0.63 g/L). Comparing to the sodium nitrate cultivation, the growth trends varied in different concentration of urea (Figure 1b). The highest peak biomass occurred at 68 h in cultivation (0.93 g/L) supplemented with 50 mM urea, followed by 30 mM (0.71 g/L). In cultivation with 15 mM and 20 mM urea, the time to peak biomass (at 0.44 g/L and 0.53 g/L) was 92 h, while in those with 5 mM and 10 mM urea the time to peak biomass (at 0.23 g/L and 0.33 g/L) was 116 h. The differential impact of SN and urea on biomass was also reported in previously published literatures as follows [20,30].

In the large-scale cultivation of filamentous cyanobacteria *Anabaena* sp. PCC 7120, sodium nitrate showed advantages over other nitrogen sources with 65% more growth [30]. Park et al. reported that Thraustochytrid *Aurantiochytrium* sp. KRS101 showed better biomass when cultivated in orange peel extract supplemented with sodium nitrate as a nitrogen source than with urea [20]. Since similar peak biomass values were obtained at 68 h in two cultivations supplemented with 50 mM SN or urea, 50 mM was chosen for further study to investigate how different nitrogen sources regulate the FA synthesis.

### 2.2. Effects of Sodium Nitrate and Urea on Fatty Acid Contents and Composition in Thraustochytriidae sp. PKU#Mn16

**Figure 2:**
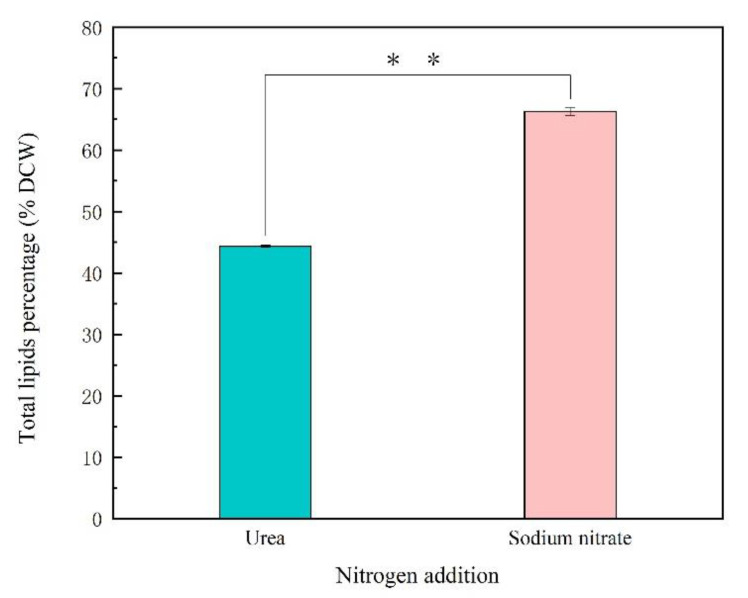
The total lipids content of *Thraustochytriidae* sp. PKU#Mn16 in 50 mM urea or 50 mM sodium nitrate cultivation. DCW represents dry cell weight. All tests were performed in biological triplicate. Values were means ± standard deviation. Statistically significant differences (*p*-value < 0.05 *) were tested by Student’s *t*-test, *p* * * <0.01.

**Figure 3:**
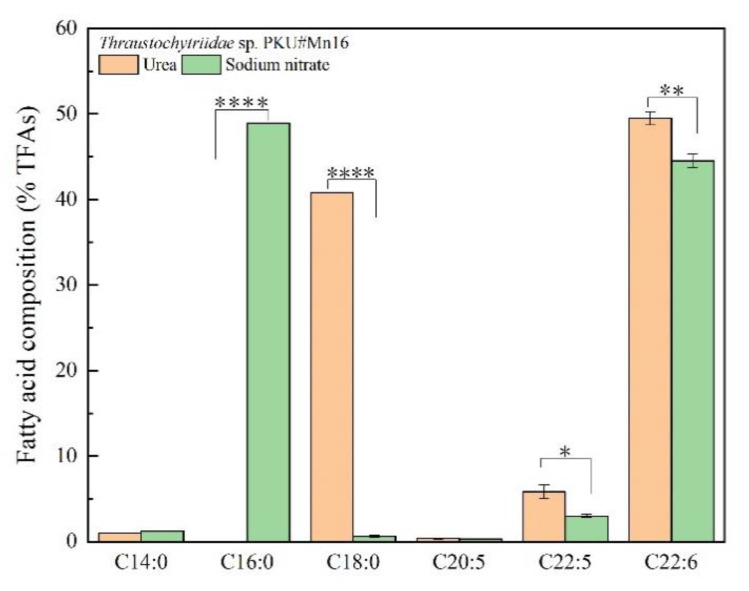
Fatty acid profile of *Thraustochytriidae* sp. PKU#Mn16 in 50 mM urea or 50 mM sodium nitrate cultivation. The name of fatty acids was shown in total carbon numbers: C14:0 = tetradecanoic acid (TDA), C16:0 = hexadecanoic acid (HDA), C18:0 = octadecanoic acid (ODA), C20:5 = eicosapentaenoic acid (EPA), C22:5 = docosapentaenoic acid (DPA), C22:6 = docosahexaenoic acid (DHA). All tests were performed in biological triplicate. Data were shown as weigh percentage of TFAs. Values were means ± standard deviation. Statistically significant differences (*p*-value < 0.5 *) were tested by Student’s *t*-test, *p* * <0.05, *p* **<0.01, *p* **** <0.0001.

To examine the effects of sodium nitrate and urea on fatty acid contents and composition for *Thraustochytriidae* sp. PKU#Mn16, 500 mg cells (dry cell weight, DCW) that cultivated in 50 mM sodium nitrate or 50 mM urea for 68 h were collected for lipids extraction. As shown in Figure 2, the total lipids extracted from SN cultivation accounted for 66.27% of DCW, which is 21.93% more than from urea cultivation (44.34% of DCW). The total lipids extracted mainly comprised six types of fatty acids, namely tetradecanoic acid (TDA, C14:0), hexadecanoic acid (HAD, C16:0), octadecanoic acid (ODA, C18:0), eicosapentaenoic acid (EPA, C20:5), docosapentaenoic acid (DPA, C22:5), and docosahexaenoic acid (DHA, C22:6) (Figure 3). The three most abundant fatty acids under sodium nitrate cultivation were HAD, DHA and DPA, whereas those under urea cultivation were DHA, ODA and DPA. It is noticeable that cells grown in the presence of urea tended to produce more n-3 PUFAs. For cells supplemented with urea, DHA was detected as the predominant composition of total fatty acids (TFAs, (49.49%)), which was 1.11-fold of that from SN cultivation (44.51%). Besides, the percentage of DPA was 5.82%, about two times of that in SN incubation (2.99%). EPA accounted for 0.37% of TFAs, also slightly higher than that derived from SN cultivation. Interestingly, a significantly high proportion (40.83%) of ODA was also observed from urea cultivation, while there was just 0.60% in their counterparts. The ratio of TDA was similar between these two kinds of samples, at 1.21% and 0.96% respectively. For cells from SN cultivation, the largest proportion of TFAs was HDA, at 48.92%, while HDA was barely detected in cells from urea cultivation. Overall, using sodium nitrate as nitrogen source benefits the production of TFAs, while urea as nitrogen source showed more advantage for n-3 PUFA accumulation.

### 2.3. Screen of the Reference Genes for RT-qPCR

To explore the molecular mechanism of how nitrogen sources affect lipids production in *Thraustochytriidae* sp. PKU#Mn16, the expression levels of key genes involved in the fatty acid metabolism pathway and nitrate metabolism pathway were quantified by RT-qPCR. However, due to the lack of available RGs for RT-qPCR normalization, it is necessary to screen novel RGs for reliable quantification. Ten putative RGs (CHIA, RPPK, NUC, MFT, CAMK1, HSF, VPS, SAC, RBATP and HTATP) were selected from the RNA-seq dataset (Table 1). Cycle threshold (Ct) value, the number of cycles that the fluorescence signal reaches the set threshold level in every reaction, is used to determine the expression levels of each gene. The higher the Ct value, the lower gene expression level, and *vice versa*. The mean Ct values of ten candidate RGs ranged from 24 to 35. The RBATP displayed the maximal Ct value of 24, while HTATP showed the minimal Ct value of 35. The Ct values of the other RGs mainly fell between 27–34. To identify optimal RGs for *Thraustochytriidae* sp. PKU#Mn16 under sodium nitrate or urea cultivation, four statistical methods (geNorm, NormFinder, BestKeeper and RefFinder) were employed to evaluate and rank the stability of their expression level (Table 2).

**Figure 4:**
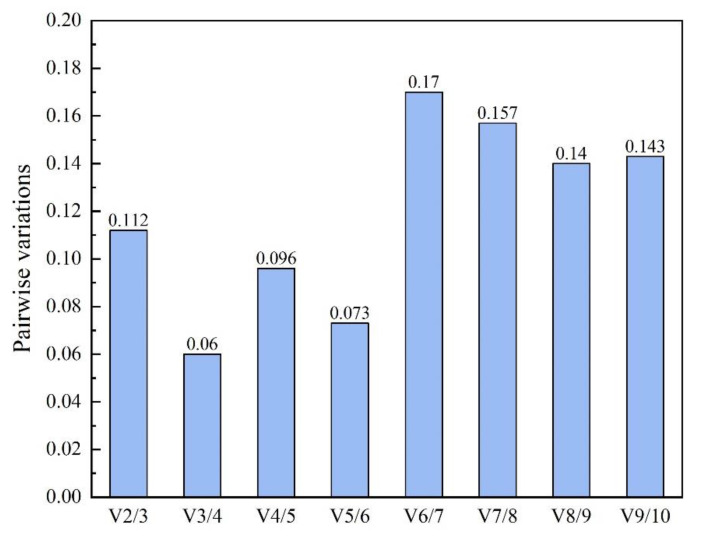
Determination of the optimal number of reference genes for normalization. Pairwise variation (V) of the candidate reference genes calculated by geNorm. To obtain reliable normalization, Vn/Vn + 1 should be smaller than a threshold of 0.15, otherwise, another reference gene (n + 1) should be added.

GeNorm analysis showed that among ten selected RGs, VPS had the lowest expression stability (M) value of 0.127, which was regarded as the best RG under the experimental condition when sodium nitrate and urea used as nitrogen sources. On the contrary, HTATP displayed the biggest M value of 0.94 and considered as the least stable RG in the same experimental treatment. Besides, the pairwise variation V_2/3_ value was 0.112 (Figure 4), which suggested that the optimal number of RG for accurate normalization was two. According to the stability value measured by NormFinder, MFT, CHIA and NUC were the best three stably expressed RGs, with stability values of 0.238, 0.315 and 0.351 respectively. The least stable RG determined with NormFinder was the same as by geNorm—HTATP, with a stability value of 1.168. The result of BestKeeper analysis showed that the most stable RG was HTATP, followed by SAC, RPPK and CHIA. VPS appeared to have the least stable expression in this algorithm. RefFinder integrates four computational programs (geNorm, NormFinder, BestKeeper and delta Ct) to compare and rank the candidate RGs. The comprehensive ranking recommended by RefFinder from the highest to the lowest stability was as follow: MFT > NUC > CHIA > HSF > VPS > RPPK > CAMK > HTATP > SAC > RBATP (Table 2). According to the comprehensive ranking of expression stability, MFT and NUC were the best two RGs and used to normalize the expression of target genes in *Thraustochytriidae* sp. PKU#Mn16 under the experimental condition of sodium nitrate and urea as the respective nitrogen source.

### 2.4. Effect of Various Nitrogen Sources on the Expression of the Key FA Synthesis Genes of Thraustochytriidae sp. PKU#Mn16

**Figure 5:**
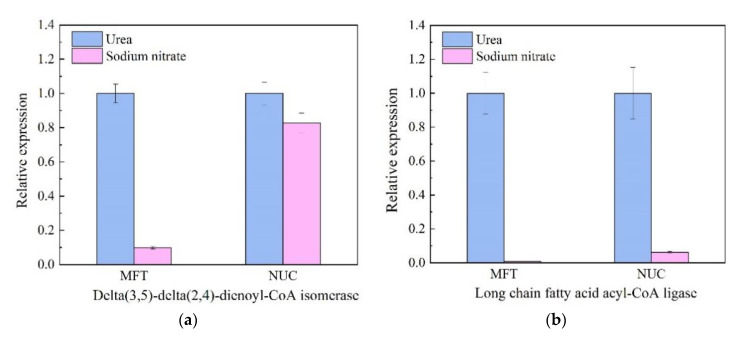

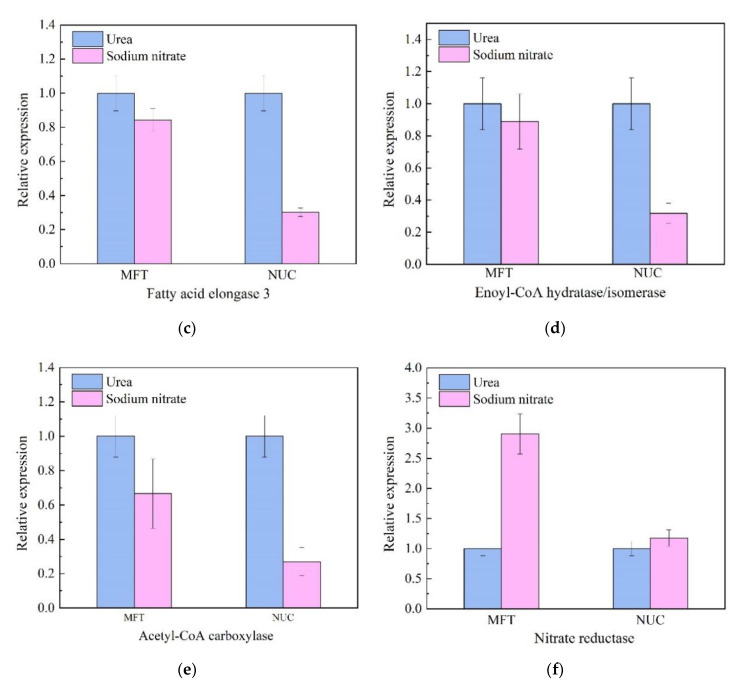
Relative expression levels of key genes involved in nitrogen metabolism and fatty acid biosynthesis using the best stable reference genes (MFT and NUC). Two best stable reference genes, MFT (mitochondrial folate transporter) and NUC (nucleolin) were selected from *Thraustochytriidae* sp. PKU#Mn16 transcriptome data and identified by four statistical methods (geNorm, NormFinder, BestKeeper and RefFinder) for stability. Relative quantification of six lipids related genes (delta(3,5)-delta(2,4)-dienoyl-CoA isomerase (**a**), long-chain fatty acid acyl-CoA ligase (**b**), fatty acid elongase 3 (**c**), enoyl-CoA hydratase/isomerase (**d**), Acetyl-CoA carboxylase (**e**) and nitrate reductase (**f**)) were normalized by reference genes MFT and NUC. The average Ct value was calculated from three biological and technical replicates and used for relative quantification of genes expression using 2^−ΔΔCT^ method. Values were means ± standard deviation.

Six genes that play important roles in fatty acid biosynthesis and metabolism were chosen to explore the mechanism on how the different nitrogen source influence the production of the fatty acids. Similar expression profiles were obtained with using either MFT or NUC as a reference gene (Figure 5), which confirmed that the methods to quantify gene expression were reliable in the present study.

Nitrate reductase gene (NR) encoding enzymes that catalyzed the first step in nitrogen assimilation pathway [31] was down-regulated in urea cultivation, as the normalized NR transcripts against MFT and NUC were 34% and 85% of those from SN cultivation respectively. However, five genes (delta(3,5)-delta(2,4)-dienoyl-CoA isomerase, enoyl-CoA hydratase, fatty acid elongase 3, long-chain fatty acid acyl-CoA ligase and acetyl-CoA carboxylase) involved in lipids metabolism were all up-regulated in urea cultivation. Delta(3,5)-delta(2,4)-dienoyl-CoA isomerase gene (DCI) played a significant role in the beta-oxidation of PUFA [13]. It was up-regulated 10.27-fold when using MFT as an RG, while up-regulated 1.20-fold using NUC. Long chain fatty acid acyl-CoA ligase gene (LCFAAC) participated in the fatty acid biosynthesis pathway [32]. The relative expression level of LCFAAC varied significantly between urea cultivation and SN cultivation, with the prominent up-regulation by urea observed (113.82-fold (MFT as RG) and 15.95-fold (NUC as RG), respectively). Fatty acid elongase 3 gene (FAE3) was an indispensable gene in fatty acid elongation [33]. When normalized against MFT, FAE3 was slightly up-regulated (1.22-fold). Enoyl-CoA hydratase gene (ECH) encoded enzyme which catalyzed a critical step of fatty acid metabolism [34]. It was up-regulated by 3.14-fold with NUC normalization. In addition, upregulation by urea was observed for acetyl-CoA carboxylase gene (ACC) (1.50-fold with MFT as RG), which encoded a key regulator in fatty acid biosynthesis. As shown in Figure 3, cells grown in urea medium accumulated more DHA, DPA, EPA and ODA than those in sodium nitrate. These five genes played significant roles in the biosynthesis of these accumulated lipids and were presumably responsible for producing significant quantities of n-3 PUFAs in *Thraustochytriidae* sp. PKU#Mn16.

## 3. Discussion

### 3.1. Essential Genes Involved in FAS and PKS Pathways of Fatty Acid Production

The biosynthesis of fatty acids in microorganism is commonly achieved by a cyclic reaction pathway by stepwise, iterative elongation with acetyl-CoA and malonyl-CoA as precursors. Fatty acid synthase (FAS) pathway mainly utilizes the large multifunctional enzyme--fatty acid synthase to catalyzes the fatty acids biosynthesis [35]. FAS harbors four catalytic domains for a respective reaction as follow: condensation, ketoacyl reduction, hydroxyacyl dehydration, and enoyl reduction [36]. In FAS pathway, saturated acid C16:0 (HDA) was formed after the cyclic FAS-catalyzed reactions using acetyl-CoA and malonyl-CoA as precursors. C16:0 was converted to C18:0 (ODA) by elongase. Then C18:0 was catalyzed by a series of desaturases (delta 9-desaturase, delta 12-desaturase, delta 15-desaturase delta 6-desaturase, delta 5-desaturase and delta 4-desaturase) and elongases to finally synthesize n-3 PUFAs, such as C20:5 (EPA), C22:5 (DPA) and C22:6 (DHA) [37,38].

Cells grown in sodium nitrate medium produced more C16:0, while those grown in urea medium produced less C16:0 but enormous C18:0. In addition, higher contents of C20:5 (EPA), C22:5 (DPA) and C22:6 (DHA) were also observed in cell cultivated in urea medium. Fatty acid elongase 3 (FAE 3) was detected in *Thraustochytriidae* sp. PKU#Mn16 and was up-regulated under urea cultivation. This indicates that under urea cultivation, a higher level of FAE gene expression was achieved, leading to more conversion of C16:0 to C18:0 and subsequently higher n-3 PUFA production by FAEs. It was reported that overexpressing the endogenous fatty acid elongase genes in *Thalassiosira pseudonana* led to the improvement of LC-PUFA production, with a 4.5-fold increase in DHA levels [39]. Hamilton et al. also found that overexpression of delta-5 elongase in diatom *Phaeodactylum tricornutum* led to an 8.0-fold increase in DHA [40]. Acetyl-CoA carboxylase (ACC) catalyzes the rate-limiting step of the FAS pathway, converting acetyl-CoA to malonyl-CoA [41,42]. In this study, the gene expression level of ACC was also up-regulated under urea cultivation, which might correspond to the higher PUFAs accumulation. It was reported that the overexpression of ACC significantly increased the conversion of acetyl-CoA to malonyl-CoA in *Aspergillus terreus* [43]. The ACC containing mutant S1157A resulted in a 3-fold increase in both polyketide and fatty acid production in yeast [44]. The Enoyl-CoA hydratase (ECH) is a homohexamer which belongs to a low sequence similarity family of CoA-binding proteins that share a hydratase/isomerase sequence motif [45]. It participated in the fatty acid metabolism process which generated acyl-CoA molecules [46]. The abundant expression of ECH in *Thraustochytriidae* sp. PKU#Mn16 under urea cultivation might translate to the generation of adequate precursors for downstream PUFA production. Long-chain fatty acid acyl-CoA ligase (LCFAAC) is an important enzyme that converts free fatty acids into fatty acyl-CoA esters, which are crucial intermediates for complex lipid biosynthesis [47]. The high expression level of LCFAAC in cells under urea cultivation might also lead to the high PUFAs production in *Thraustochytriidae* sp. PKU#Mn16.

The polyketide synthase (PKS) pathway synthesized lipids through repetitive cycles of four principal reactions: condensation by ketoacyl synthase (KS), ketoreduction by ketoreductase (KR), dehydration by dehydratase (DH), and enoyl reduction by enoyl reductase (ER). An isomerase was in charge of the conformation of the fatty acid chain [48]. However, this pathway was reported to be lack of complete enzymes in *Thraustochytrids* [49]. In our genome sequencing annotation of *Thraustochytrium* sp. SZU445 [13], KS, KR and ER was detected, but DH and isomerase were not identified. However, delta(3,5)-delta(2,4)-dienoyl-CoA isomerase (DCI) that catalyzed the isomerization of 3-cis-octenoyl-CoA to 2-trans-octenoyl-CoA was detected, and it was regarded as the isoenzyme of DH and isomerase in PKS pathway, which lead to the transpose of the unsaturated carbon bonds to the correct sites. In the urea cultivation, a higher level of DCI gene expression was observed, probably contributing to the formation of the carbon-carbon double bonds in DHA and EPA. Rai et al. found that overexpression of human peroxisomal enoyl-CoA delta isomerase2 HsPECI2 changed the polar lipid content of tobacco. The production of phosphatidylcholine, phosphatidylserine and digalactosyldiacylglycerol were moderately upregulated [50].

### 3.2. Effects of Nitrate Reductase on Nitrogen Assimilation and Lipids Production

Nitrogen is one of the essential components in living organisms due to its participation in the construction of biomolecules such as proteins and nucleic acids [51]. Previous studies have shown that *Thraustochytrids* harbor the ability to utilized nitrate and a variety of nitrogen-containing compounds as nitrogen sources (Table 3). The preferable and non-preferable nitrogen source for *Thraustochytrids* is species specific. However, most of the previous study mainly focused on the fermentation optimization by using various nitrogen sources, the molecular mechanism that contributes to the nitrogen source dependent PUFA production was seldom explored.

In the nitrate assimilation pathway, nitrate reductase (NR) plays an important role and can be found in bacteria, microalgae, fungi and plants [51,54,55]. NR is a cytosolic enzyme that catalyzes the very first step in nitrogen assimilation, that is, to convert nitrate (NO_3_^−^) to nitrite (NO_2_^−^). The nitrite is then catalyzed by nitrite reductase to ammonium (NH_4_^+^) [56]. It has been demonstrated that the activities of NR are high in nitrate cultivated cells and low when ammonium used as a nitrogen source to cultivate cells [57]. Urea is an economical friendly organic nitrogen source. In the urea metabolism, urease, one of the extracellular enzymes of *Thraustochytrids* [58], catalyzes the conversion of urea to carbon dioxide and ammonia. Interestingly, in the present study the gene expression of NR was down-regulated in *Thraustochytriidae* sp. PKU#Mn16 under urea cultivation compared to that under sodium nitrate cultivation, which was consistent with NR activities. In the study of Benhima et al., sodium tungstate was used to inhibit NR in microalgae *Dunaliella tertiolecta*, resulting in a 50% increase of neutral lipids. Besides, fatty acid methyl esters composition showed a slight variation of polyunsaturation and elongation [59]. McCarthy et al. found that the knockout (KO) of NR gene impacted the lipids metabolism in model pennate diatom *Phaeodactylum tricornutum*. The concentration of triacylglycerol was increased in NR-KO cells, consistent with up-regulated key genes of triacylglycerol biosynthesis [56]. Consistent with published works, our results showed sodium nitrate induced a relatively high expression level of NR, and urea down-regulated NR expression resulting in more long chain PUFAs.

## 4. Materials and Methods 

### 4.1. Microorganism Cultivation 

*Thraustochytriidae* sp. PKU#Mn16 was preserved in China General Microbiological Culture Center (CGMCC) under the accession nos. 8095. Modified artificial seawater [60] (0.05 g/L KH_2_PO_4_, 0.6 g/L KCl, 2.44 g/L MgSO_4_, 0.3 g/L CaCl_2_.2H_2_O, 1 g/L Tris-HCl (pH 8.0), 10 mL/L PI metal, 3 mL/L chelated iron solution, 18 g/L NaCl and 20 g/L glucose dissolved in deionized water) was used to prepare nitrogen addition medium. 2 M sodium nitrate solution and urea solution were prepared and added into the modified artificial seawater respectively to make a series of concentrations of nitrogen medium: 1 mM, 5 mM, 10 mM, 15 mM, 20 mM, 30 mM and 50 mM. Cultures were then incubated on an orbital shaker (LYZ-123CD, Longyue Company, Shanghai, China) at 23 °C, 200 rpm for 140 h. Three parallel cell samples were collected from each medium at regular intervals to analyze biomass and generate growth curve. Cells were washed three times with deionized water and centrifugated (Z366K, HERMLE, Wehingen, Germany) at 25 °C, 7871× *g* for 6 min, then cell pellets were lyophilized in a freeze dryer (Triad 2.51, Labconco, Kansas City, MO, USA) to calculate dry cell weight (DCW).

### 4.2. Lipids Extraction and Fatty Acid Composition Analysis

Lipids of three biological replicates were extracted by the modified Bligh and Dyer procedure [61]. 500 mg freeze-dried cells were extracted with a solvent mixture of chloroform/methanol (1:2, *v*/*v*) in a Soxhlet extractor at 70 °C for 96 h. Crude total lipids were gained after the solvent mixture was evaporated. Fatty acid methyl esters (FAMEs) were prepared by a direct acid-catalyzed transesterification [62,63]. Crude lipids were added to 4 mL 4% sulfuric acid in methanol and incubated at 70 °C for 1 h. Subsequently, FAMEs were extracted in 2 mL n-hexane and 2 mL deionized water. After the upper organic layer was transferred to a new tube and dried with nitrogen stream, 1 mL dichloromethane was added and the whole solution was transferred to a chromatography bottle, ready for gas chromatography-mass spectrometry (GC-MS, 7890-5975 Agilent, Santa Clara, CA, USA) analysis. 

In the GC-MS analysis, GC column used for the determination of FAMEs is HP-5MS (19091S-433, 30.0 m × 250 μm, I.D. × 0.25 μm film thickness, Agilent J&W) with a maximum temperature of 350 °C. An aliquot of 1 μL of each sample was injected into the GC column. The inlet temperature of GC was set to 250 °C. High purity Helium was used as the carrier gas. Constant pressure mode was used, and the split ratio was 10:1. The temperature program was set as follows: first, the temperature is raised from 60 °C at a rate of 25 °C/min to 180 °C, then the temperature was raised to 240 °C at a rate of 3 °C/min, holding at 240 °C for 1 min, and then raised to 250 °C at a rate of 5 °C/min. The GC-MS transfer line temperature was set to 250 °C, and the full scan mode was employed for the GC-MS detection. A 37-component fatty acid standard mixture was used to validate the temperature program that separated sequentially the peaks of 37 fatty acids. Nonadecanoic acid was used as an internal standard and the content of the fatty acids was quantified by comparing the peak areas of that of the internal standards. Three biological replicates were examined.

### 4.3. RNA Extraction and cDNA Synthesis

Cells were collected (7871× *g* for 6 min) after cultivation for 68 h. Cell pellets were then washed three times with distilled water and subsequently frozen at −80 °C for 2 h. The frozen cell pellets were ground in liquid nitrogen to fine powder then 100 mg powder was transferred into a clean 1.5 mL centrifuge tube. Subsequently 1 mL TRIzol (Life Technologies, Carlsbad, CA, USA) reagent was added into the tube and set in room temperature (RT) for 30 min, after that 0.2 mL chloroform was added and the mixture was vortexed for 30 s, rest in RT for 5 min then centrifuged at 17,709× *g* for 15 min. The upper layer was transferred to a new centrifuge tube and was added 0.5 mL isopropanol and placed in RT for 10 min. Total RNA precipitate was obtained after centrifugation at 17,709× *g* for 15 min at 4 °C, and then it was washed twice with 75% ethanol and dissolved with DNase/RNase free water. The integrity of RNA was checked by 1.5% (*w*/*v*) agarose gel electrophoresis. The quality and concentration of RNA were determined using a NanoDrop-2000 spectrophotometer (Thermo Fisher Scientific Inc., Waltham, MA, USA). Total RNA with 260/280 nm ratio of 1.9–2.2 and 260/230 nm ratio greater than 1.9 was used to synthesize cDNA with PrimerScript^TM^ RT reagent Kit (Takara, Japan) according to the manufacturer’s guidelines. The cDNA was stored at −20 °C for later use.

### 4.4. Selection of Candidate Reference Genes from RNA-Seq Data

Candidate reference genes (RGs) were selected from the RNA-seq dataset of *Thraustochytriidae* sp. PKU#Mn16 under nitrogen addition cultivation [15]. The coefficient of variation (CV) of FPKM (Fragments Per Kilobase of transcript per Million mapped reads) was calculated across all the treatments and genes with lower CV values were considered stably expressed. Candidate RGs were chosen under the following requirements that the CV of FPKM was less than 10% and annotation of genes was adequate.

### 4.5. Real-Time Quantitative Polymerase Chain Reaction (RT-qPCR)

The primers used for real-time quantitative PCR (RT-qPCR) were designed according to transcriptomic sequence data using Primer Premier 5 software. Primers were synthesized with the following parameters: Primer length of 18–25 bp, product lengths of 80–200 bp, Tm values of 50–65 °C and GC content of 45–55%. RT-qPCR reactions were performed in the QuantStudio 6 Flex Real-Time PCR System (ABI, Vernon, CA, USA). The SYBR TaqTM ExPremix (Tli RNaseH Plus) Kit (Takara, Shiga, Japan) was used with the following cycling conditions: initial denaturation at 95 °C for 30 s; 40 cycles of 95 °C for 5 s and 60 °C for 30 s. The programs SDS v2.4 (ABI, Vernon, CA, USA) were used for monitoring the qPCR reactions and for analyzing primer efficiencies, R^2^ of efficiencies and gene expression level. To confirm primer specificity, only melting curves of amplicons presenting single peaks were selected for further analysis. Relative gene expression was quantified using the 2^−^^△△CT^ method [64]. Three biological and three technical replicates were performed in all RT-qPCR analysis.

### 4.6. Assessment of Gene Expression Stability 

Four statistical programs, geNorm, NormFinder, BestKeeper and RefFinder, were used to assess the stability of the RGs across all experimental conditions. The geNorm program calculates the gene expression stability (M) value for each gene. Genes with an M value less than 1.5 are considered to be stably expressed, the lower M value the more stable expression. In addition, this program also calculates a pairwise variation (Vn/n + 1) value between genes. The value of “n” is the optimal number of RGs when the pairwise value of variation (Vn/n + 1) is below a cut-off value of 0.15. The NormFinder program evaluates the stability of candidate RGs by an assessment of within and between-group variations. A lower variation value indicates a better reference gene. The BestKeeper program examines the coefficient of variation (CV) and standard deviation (SD) based on the raw Ct values of all candidate RGs. Any gene with an SD value lower than 1 was considered as a gene with stable expression. The most stable RG recommended by BestKeeper program is the one with both the lowest CV and SD values. The Delta Ct approach compares the difference in Ct values of RG pairwise and ranks the candidate RGs using the variability of averaged SD. RefFinder (https://www.heartcure.com.au/reffinder/) integrates the four algorithms (geNorm, NormFind, BestKeeper and delta Ct) methods. The stability of candidate RGs was comprehensively validated and ranked by calculating the geometric mean of their weights for the overall final ranking.

### 4.7. Validation of Reference Genes

Six genes (delta(3,5)-delta(2,4)-dienoyl-CoA isomerase, enoyl-CoA hydratase, fatty acid elongase 3, long chain fatty acid acyl-CoA ligase, acetyl-CoA carboxylase and nitrate reductase ) which was annotated in RNA-seq of *Thraustochytriidae* sp. PKU#Mn16 as responsible genes for lipid metabolism were used to verify the stability of selected RGs. The top two best RGs were used to normalize the expression of target genes in the present study. The RT-qPCR thermocycling protocol was the same as described above.

### 4.8. Statistical Analysis

Statistical analysis of the experimental data was performed by using Students’s *t*-test in the GraphPad Prism 8.0.1 software, and the statistical difference amongst groups was determined by the *p* value at *p* *< 0.05, *p* * * < 0.01, *p* * ** < 0.001, *p* * ** * < 0.0001 [65].

## 5. Conclusions

In the present study, sodium nitrate as a nitrogen source is beneficial to the short-chain fatty acids’ accumulation in *Thraustochytriidae* sp. PKU#Mn16, while urea as nitrogen source has more advantage for n-3 PUFA biosynthesis. Out of ten reference genes, two genes (MFT and NUC) showed higher expression stability and were chosen for RT-qPCR normalization. Five essential genes related to the long-chain fatty acid synthesis encompassing (delta(3,5)-delta(2,4)-dienoyl-CoA isomerase, enoyl-CoA hydratase, fatty acid elongase 3, long-chain fatty acid acyl-CoA ligase and acetyl-CoA carboxylase) were up-regulated in the cultivation using urea as the nitrogen source, while nitrate reductase was up-regulated in sodium nitrate cultivation. These findings shed light on the underlying molecular mechanisms of n-3 PUFA production in *Thraustochytrids* and revealed the key genes which could be exploited in the improvement of n-3 PUFA production.

## Figures and Tables

**Table 1:** Candidate reference genes and primer sets for RT-qPCR (real-time quantitative polymerase chain reaction).

Gene Name	Gene Symbol	Primer Sequence (5′–3′) (Forward/Reverse)	Product Size (bp)	RT-qPCR Efficiency (%)	R^2^
Chitinase	CHIA	GACCCGCTCACCTACTTCAA	118	101.616	0.998
		TCTGTCATCTGCTGCTCCAC			
Ribose-phosphate pyrophosh-okinase	RPPK	GAGGCCGTGGGTAGTAAAGG	172	98.02	0.996
		GGATTGGACTGAGGAGGAGC			
Nucleolin	NUC	GACGAGCGTGAACTTGAGCG	106	109.201	0.994
		AACCAGACGAAGAGGACGAG			
Mitochondrial folate transporter	MFT	ACACTACCGCAGCCTATCAC	113	97.816	0.995
		ATCCATCTGTCAAGCCATCC			
Calcium dependent protein kinase I	CAMK1	CACCAGAACGGCATCATCCA	154	95.226	0.997
		GACCAAAGTCACAAAGCACC			
Heat shock transcription factor	HSF	TCCCTTCAGTTTCACCACAT	192	102.598	0.998
		AAGCACCCACTATTCCAACG			
Vacuolar protein sorting-associated protein	VPS	CCCCAGAAGGAAACCATCAC	164	102.201	0.996
		TTCATCGCACAGCAGTAGGC			
Saccharolysin	SAC	TAAGGGTCCAAGAAGAATGA	100	99.577	0.996
		CTGACGGCGAAGTTCCTGTG			
Ribosome-binding ATPase	RBATP	CCTTGGGCATGTCTACTTCT	126	97.341	0.998
		GTCTGAAACGAGCGAACACC			
H^+^-transporting ATPase	HTATP	TATCCAACCGTAGCCACAGA AGGGTTTCGAGTAGGAGTGC	123	102.301	0.998

The RT-qPCR amplification efficiency and the R^2^ of efficiency for each primer were determined by QuantStudio Software V1.2.4. The range of RT-qPCR efficiency should be 90–110%, and R^2^ should be greater than 0.98.

**Table 2:** Ranking of the expression stability of 10 candidate reference genes (ranking order: Better-Good-Average).

Method	1	2	3	4	5	6	7	8	9	10
Delta Ct	MFT	NUC	HSF	CHIA	CAMK	RPPK	VPS	SAC	RBATP	HTATP
BestKeeper	HTATP	SAC	RPPK	CHIA	RBATP	MFT	NUC	HSF	CAMK	VPS
NormFinder	MFT	CHIA	NUC	HSF	CAMK	RPPK	VPS	RBATP	SAC	HTATP
geNorm	VPS	HSF	CAMK	NUC	MFT	CHIA	RPPK	SAC	RBATP	HTATP
Comprehensive ranking	MFT	NUC	CHIA	HSF	VPS	RPPK	CAMK	HTATP	SAC	RBATP

**Table 3:** Effects of different nitrogen sources on lipids production of *Thraustochytrids*.

Nitrogen Source	Species	Culture Time	Culture Mode	Lipid Content (%)		References
				TFAs (% DCW)	DHA (%TFAs)	
Orange peel extract & sodium nitrate	*Aurantiochytrium* sp. KRS101	108 h	baffled flasks	50.23%	28.51%	[20]
Yeast extract	*Schizochytrium* sp. PQ6	96 h	10 L fermenter	38.67%	43.58%	[52]
Monosodium glutamate	*Aurantiochytrium* sp. SW1	96 h	Shake flasks	79.60%	47.90%	[53]
Ammonium nitrate	*Aurantiochytrium* sp. SW1	96 h	Shake flasks	46.76%	31.25%	[53]
Sodium nitrate	*Thraustochytriidae* sp. PKU#Mn16	68 h	Shake flasks	66.27%	44.51%	This study
Urea	*Thraustochytriidae* sp. PKU#Mn16	68 h	Shake flasks	44.34%	49.49%	This study

TFAs: total fatty acids; DHA: docosahexaenoic acid; DCW: dry cell weight.
